# Therapeutics

**Published:** 1859-03

**Authors:** 


### THERAPEUTICS.

1. LIGATURE OF THE EXTREMITIES IN INTERMITTENT FEVER. By *Drs. J. De Brauw* and *H. J. Brœrs.*—According to Drs. De Brauw and Brœrs, the ligature of the extremities is a measure which has been already employed by ancient physicians to aid in the treatment of intermittent fever, but has unjustly nearly fallen into oblivion. Already Pinius (Hist. Nat. xxviii., 6,) knew this antiperiodic, as Pittschaft (Hufeland's Journ., li., 3, pp. 47, 48,) states, and in Van Swieten's *Commentaries to Boerhaave's Aphorisms* the "levis brevisque compressio venarum in artubus," is strongly recommended as a means to relieve the burning heat of fever. Dr. V. Hildenbrand, however, declares the remedy, in his *Institutiones Practico-Medicæ*, to be unreliable, and in many respects unsafe, and recommends caution in the use of it. Jos. Frank (Prax. Med. Univ. Præcepta) speaks of it in a very superficial manner, like many other, particularly more recent authors. One of the most enthusiastic commenders of this method is George Kellie, (Duncan's Medical Commentaries, vol. xix.,) who, during the siege of Willemstad by the French army, in 1793, cured many cases of intermittent fever (which had resisted the use of quinine) completely, by compression of the extremities. Upon his recommendation several physicians in England—for instance, Veitch and Wallich, (Mediz. Nationalzeitung, July, 1798,) and in the Netherlands, (Agemene Vaderl. Lettervefningen, 1808, 5,)—tried his method with signal success. Of the more recent communications on this subject, that of Prof. Chladni (Hufeland's Journal, xlii. p. 133,) is worth particular attention. This celebrated savant being attacked in 1813 by an obstinate intermittent fever, used the remedy with much advantage. He describes it as quite innocuous, and explains its curative influence by the supposition, that by the ligature of the extremities the return of blood to the heart, and to the centres in general, is hindered or partially suspended, and that the full development of one of the principal symptoms, the chill, being thus interfered with, an interruption and disturbance of the whole type of fever takes place.

This method belongs moreover to one of the oldest popular remedies used in Russia, England, and France. In *Canstatt's Jahresbericht*, (Jaborg., 1848, p. 113,) the cure of a quartan by application of Junod's boot is mentioned, a fact which seems to be intimately connected with the subject in question. According to Jolly, (Dict. de Méd. et de Chir., tome xi. part i. p. 363,) who gives a detailed account of the *ligatures circularies des membres*, the ligature should be applied to the four extremities at the same time, but in such a manner that only the circulation in the superficial vessels is suspended. Martinet, Robinau, Récamier, and Husson, kept up the compression for not longer than twenty-five to fifty minutes, and commenced with it in the cold stage. Jolly recommends taking off the ligatures one by one, at intervals of several minutes, as by the simultaneous removal of the same too much blood would be at once introduced into the circulation, which might be attended with evil consequences.

The most complete information on the subject of his investigation the author found in a dissertation of R. v. Bærle: "*De valde multiplici febrium intermittentium medicatione speciatim de membrorum majorum circumstrictione tantaminibus in nosocomio academico explorata*," Utrecht, 1809. In this treatise the ligature of the extremities is thoroughly illustrated by the report of seven cases, and highly recommended. V. Bærle commenced the treatment with the administration of a gentle purgative; the patients were kept in bed, and subjected to a rigid diet during the paroxysm; shortly before the commencement of the cold stage, the thighs and upper arms were encircled by ligatures exercising a moderate pressure, which were removed in from six to fifteen minutes, or later, according to the effect they produced; after Wallich's example he forbade warm drinks during the cold stage, but recommended cold drinks in the hot stage. From observations of this kind the author draws the following conclusions: The ligature of the extremities is a safe and powerful means of assistance in the treatment of intermittent fever; it is not only an adjuvant to other antiperiodics, but also a febrifuge by itself. It cures the febris intermittens simplex and duplex, as well as the quotidiana. In regard to the quartana no experience has been made. The ligatures must be allowed to remain until the hot stage begins; a longer application does not lessen their effect. The method seems to owe its curative property to the disturbance of the usual course of the fever, (Chladni.) Sometimes the paroxysm is transferred under this treatment from the third day to the second, but generally so that the tertian type is not interrupted, or that a febris duplex is developed. The compression of the extremities is always followed by some increase of the heat and perspiration, the signs of an energetic reaction. After repeated use of this method the fever gradually subsides. Contra-indications to it never existed, but may be easily inferred from an examination of the modus operandi of the remedy.

Dr. De Brauw generally applied compression to two extremities only, but considers the ligature of all four far more efficacious in obstinate cases, and recommends the method as being capable in some cases to substitute the use of quinine.

In cases of relapse of intermittent fever, in which the patients complain of that characteristic pain in the lumbar region, (fifth lumbar vertebra,) against which cups are used without effect, Brœrs recommends the application of the galvanic current to the mentioned spot as a highly serviceable, though occasionally inefficient means. After the second or third application of this remedy the cachectic appearance, as well as the depressed feelings of the patients, underwent a favorable change. Relapses of the fever, consequent upon a return of the patient into the malarious district, yielded quickly to this mode of treatment, even when quinine was administered without success.—(*Nederland. Tijdschr.*, 1858, and *Medizinische Neuigkeiten*, 1858, No. 44.)

